# Implementation of Disaster PrepWise, an Online Disaster Preparation Tool for Older Adults

**DOI:** 10.1093/geroni/igaa057.1557

**Published:** 2020-12-16

**Authors:** Lena Thompson, Emily Henja, Travis Beckman, Sato Ashida

**Affiliations:** 1 University of Iowa College of Public Health, Iowa City, Iowa, United States; 2 Rush University, Chicago, Illinois, United States; 3 ^3^ Johnson County Homeland Security & Emergency Management Agency, Iowa City, Iowa, United States

## Abstract

Older adults are less prepared for disaster situations than younger adults due to expenses, complicated preparation processes, and lack of support. We previously developed and tested a disaster preparedness program for older adults and showed its impact on improving preparedness and personal emergency support network. Using a 5-step community-engaged approach and in collaboration with our Stakeholder Advisory Board (SAB), we adapted this Disaster PrepWise program to an online platform and developed implementation plans within existing community-based service infrastructure. Community engagement processes allowed us to create an online tool that was considered “important” and “useful” by older adults and program deliverers. In terms of reach, 73 potential participants attended an introductory presentation and 47 participated. Those who participated were similar to those who did not in terms of gender and race. Twenty-seven participants completed surveys before and one-month after the program and reported engaging in an average of 2.9 additional recommended behaviors after intervention (t(26)=2.85, p=0.00). The major organizational barrier to adoption was limited staff time. Participating older adults reported adopting what they learned through the program in their daily life. Implementation process data showed multiple modifications made during delivery including delivery of the program in one instead of two sessions. The implementing organization reported their likelihood of maintaining this program within their practice as “extremely likely.” Community-engaged approaches and process data led to an acceptable and useful program and implementation strategies that helped enhance preparedness behaviors among older adults. Future work will focus on program dissemination and larger scale evaluation.

